# Pyripherals: A Python Package for Communicating with Peripheral Electronic Devices

**DOI:** 10.21105/joss.04762

**Published:** 2022-11-10

**Authors:** Abraham Stroschein, Ian Delgadillo Bonequi, Lucas J. Koerner

**Affiliations:** 1 Department of Electrical and Computer Engineering, University of St. Thomas

## Statement of Need

We are developing a data acquisition system (DAQ) for real-time feedback that uses FPGA-based control of and acquisition from various electronic chips, or peripherals. Because these peripherals communicate over multiple protocols (SPI, I2C, LVDS) through an FPGA, we designed *pyripherals* to organize and abstract registers, the communication protocol, and the host computer interface to each communication controller. The software and firmware are designed for Opal Kelly FPGA modules, yet the Python developments are generally useful to organize communication with peripheral chips.

Data from a register is accessed with the address of the register and the bit indices of the data field, but users will often refer to the data field by its name, which *pyripherals* retains as a dictionary key. When passed the location of the data field, parameterized functions automatically format the data or command as required by the communication interface used by the peripheral. The assembled message is passed to the appropriate hardware controller responsible for low-level communication with the peripheral. In this solution, the addressing, bit indexing, and formatting are handled by *pyripherals* before the message is sent over the Opal Kelly FrontPanel API to a hardware-level communication controller on an Opal Kelly FPGA (“FrontPanel^®^,” 2022), which handles low-level communication with the desired peripheral.

This DAQ system and the *pyripherals* software are crucial components of a digital feedback amplifier being developed for ion channel electrophysiology (Koerner et al., 2022) and more generally comprise an open-source system for microsecond latency real-time feedback experiments. Similar DAQ systems (Leibrandt & Heidecker, 2015; Yu et al., 2018) create MHz bandwidth servos for physics experiments but the host software is either not publicly available or not generalizable. Many Python packages for lab automation and electrical instrument control have been developed (Allan et al., 2019; Casagrande, 2020; Jermain & Rowlands, 2020) yet do not support real-time control. In our system, the FPGA controller fills a need for microsecond level latency by interfacing directly to analog-to-digital converters and digital-to-analog converters while *pyripherals* organizes the configuration of these chips. The *pyripherals* software can be expanded to control and read data from other electronic sensors, such as accelerometers, and is currently in use in our lab with a time-of-flight depth sensor (AMS TMF8801) for rapid readout of photon return time histograms. Similar to *pyripherals*, the Python package *registerMap* (Smiley, 2019) creates a framework for register map organization in embedded systems. However, it lacks the interconnection between register data and hardware communication controller offered by *pyripherals*. The Opal Kelly XEM7310 FPGA that we use for communication controllers to demonstrate *pyripherals* is common in research environments such that our *pyripherals* software may accelerate developments of FPGA to electronic chip interfaces in other labs.

## Summary

### Registers

*pyripherals* reads an Excel spreadsheet that holds the name, address, lower bit index, upper bit index, and bit width of each data field. Different peripherals can be separated into different sheets within the register index. The table below shows an example sheet for a peripheral named “MyADC”.

- Name = name of the data field chosen by the user. This is how the register will be accessed in Python.
- Hex Address = address of the register on the chip as given by its datasheet. Must be formatted as a hexadecimal number with “0x” prefix.
- Default Value = the default value of the register as given by the chip’s datasheet.
- Bit Width = the number of bits in the data field.
- Bit Index (High) = the bit index of the upper end of the data field in the register. Ex. in a 32-bit register where the data field is located in the last 4 bits of the register, Bit Index (High) would be 31.
- Bit Index (Low) = the bit index of the lower end of the data field in the register. Ex. in a 32-bit register where the data field is located in the last 4 bits of the register, Bit Index (Low) would be 28.

*pyripherals* then reads the spreadsheet, referred to as a register index in the documentation, and returns a dictionary of name-Register pairs using the Register.get_chip_registers static method. The example below retrieves the register index from the table above.


»> MYADC_regs = Register.get_chip_registers(‘MyADC’)

Each Register object holds all values from the spreadsheet for the data field it represents. A guide for creating a register index is located in the documentation. An example register is shown below.


»> print(MYADC_regs[‘RESULT’])
0×0[0:11]
»> MYADC_regs[‘RESULT’].__dict__
{‘address’: 0, ‘default’: 15, ‘bit_index_high’: 11, ‘bit_index_low’: 0, ‘bit_width’: 12}


The register abstraction of *pyripherals* allows user code to refer to data fields using only their names. The spreadsheet organization of data fields allows for user-friendly editing and sharing of data field information without the need to change user code. Specific applications include communicating with microcontrollers or development boards like Arduino as well as accessing data using SPI or I2C controllers.

*pyripherals* uses registers to assemble messages for SPI (Srot, 2016) and I2C (I2CController, 2021) communication interfaces using parameterized commands in classes for SPI, hardware-timed SPI, and I2C. Communication specific to an individual peripheral is available in its subclass. A full list of peripherals with classes in *pyripherals* can be found in the documentation.

### Endpoints

For our research, *pyripherals* is paired with hardware controllers instantiated on an Opal Kelly FPGA. These controllers send communication signals to the chips connected to the FPGA. To interact with these controllers, *pyripherals* uses the Opal Kelly FrontPanel API for bidirectional communication over USB between a host computer and the FPGA using addressable endpoints.

*pyripherals* reads FrontPanel endpoint addresses and bit indices from a Verilog definition file. This Verilog file requires a naming system described in the documentation and associates peripherals with a specific hardware controller instantiation. A complete guide to creating an endpoint definitions file is in the documentation. Each endpoint definition has an address offset, a bit-field width, and an amount to increment the address if a second or subsequent instance is generated. The line below shows an example that defines an endpoint named “WRITE_IN” that belongs to peripheral “MYADC” with an address of 0×04 (the 8’h prefix below is Verilog syntax that indicates an 8-bit hexademical number) and a bit_width of 32 that adds 7 to the address every time it is advanced.


`define MYADC_WRITE_IN_GEN_ADDR 8’h04 *// bit_width=32 addr_step=7*


The naming convention for endpoints that contain addresses or bit indices is demonstrated below with curly brackets {} indicating placeholders to be completed by the user. Endpoint directions are from the perspective of the FPGA so *WRITE_IN* is data from the host computer into the FPGA that is destined for the ADC chip. More information on the syntax and meaning of these lines is available in the endpoint definitions guide.

Addresses:


`define {CHIPNAME}_{ENDPOINT_NAME}{_GEN_ADDR} {hexadecimal address} 
// bit_width={bit_width} addr_step={addr_step}


Bit Indices:


`define {CHIPNAME}_{ENDPOINT_NAME}{_GEN_BIT} {decimal bit index} 
// addr={address or endpoint name} bit_width={bit_width}


Note: the above comments must be placed on the same line as the ‘define. They are split here for readability.

For multiple units of the same chip, each chip class has a create_chips method which instantiates a specified number of chips, incrementing the endpoint addresses and bit indices according to the GEN_ADDR, GEN_BIT, bit_width, and addr_step parameters above.

Once created, the user can read the endpoint definitions file with endpoint.get_chip_endpoints which returns a dictionary of name-Endpoint pairs. An example using the “MYADC_WRITE_IN” endpoint from earlier is shown below.


»> MYADC_eps = Endpoint.get_chip_endpoints(chip_name=’MYADC’)
»> print(MYADC_eps[‘WRITE_IN’])
0×4[None:None]
»> MYADC_eps[‘WRITE_IN’].__dict__
{‘address’: 4, ‘bit_index_low’: None, ‘bit_index_high’: None, ‘bit_width’: 32, ‘gen_bit’: False, ‘gen_address’: False, ‘addr_step’: 7}


The endpoint class in *pyripherals* extends the capabilities of the Opal Kelly FrontPanel API by automatically linking the Python and Verilog endpoint data with a shared definitions file. With *pyripherals*, when the user changes the value of an endpoint in the definitions file the change is reflected in both the Python and Verilog code.

## FPGA Data Acquisition Code

Our FPGA code for use with *pyripherals* is available at https://github.com/lucask07/covg_fpga/. It is written for the Opal Kelly XEM7310 FPGA and supports I2C, SPI, and LVDS communication with a DDR for data buffering. An example use of this code is an impedance analyzer using a DAC80508 digital-to-analog converter (DAC80508, 2018) and an ADS8686 analog-to-digital converter (ADS8686S, 2020) communicating over SPI.

### Links

Documentation is available at https://pyripherals.readthedocs.io/en/latest/index.html and the GitHub is available at https://github.com/Ajstros/pyripherals. *pyripherals* is available for install from pip at https://pypi.org/project/pyripherals/.

## Current Research

*pyripherals* was developed under an NIH-funded project to create a digital ion channel amplifier at the University of St. Thomas where it is being used to communicate with and control an FPGA-based data acquisition system for real-time feedback.

## Figures and Tables

**Fig. 1:**
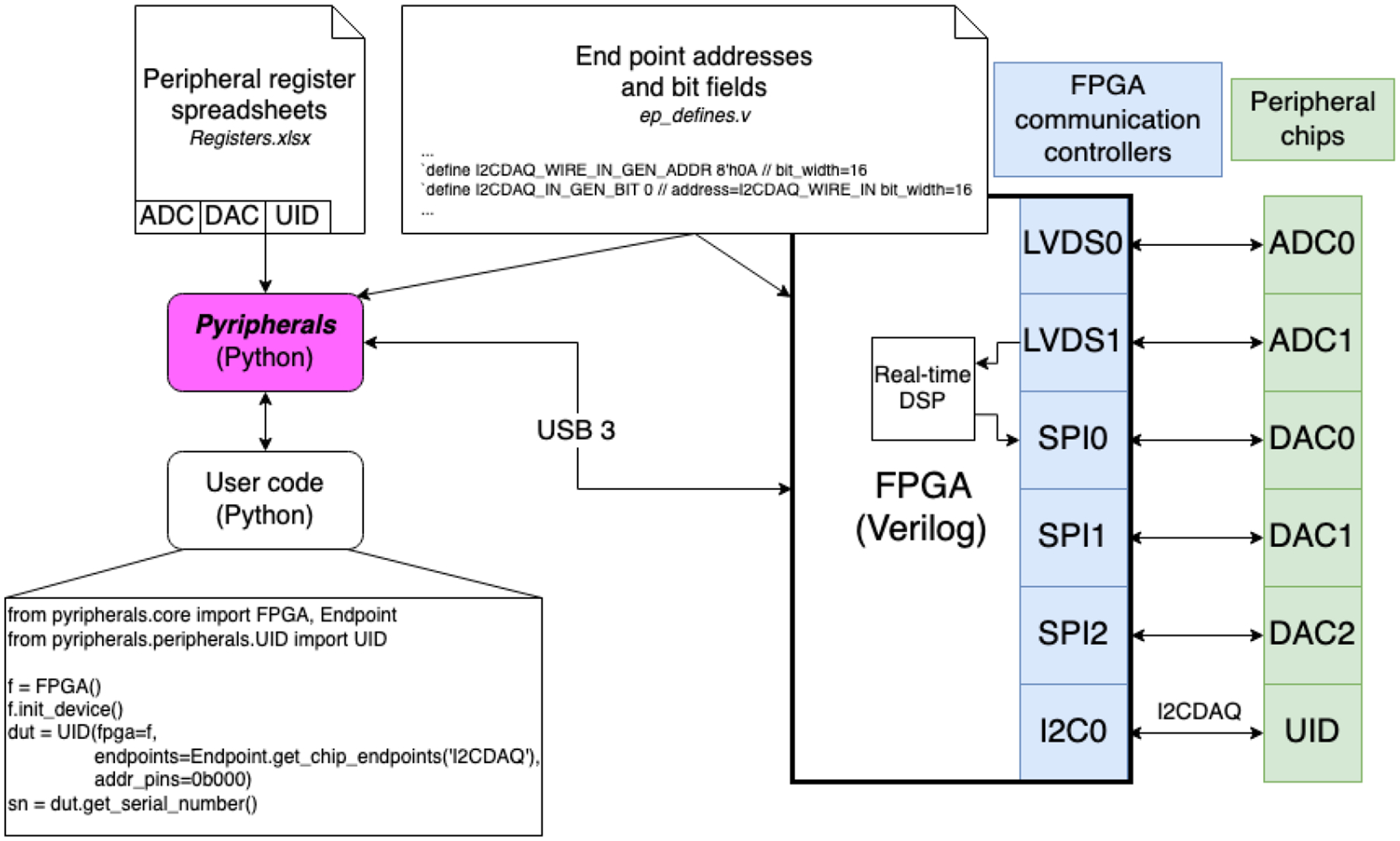
On the left, the pyripherals module abstracts registers to enable user code to easily read and write to peripherals. pyripherals and the FPGA code share a file (ep_defines.v) that defines the addresses of endpoints so that the transport of messages from the host computer through the FPGA and to the correct peripherals is managed. On the right, is an example hardware setup supported by Pyripherals. An FPGA module (Opal Kelly) with a Python API to the USB interface has various types of communication controllers in the FPGA logic and is wired to peripheral chips that sit on a custom circuit board. The chips include analog-to-digital converters (ADCs), digital-to-analog converters (DACs), and a unique identification chip (UID). The example user code reads the serial number of the UID chip using get_serial_number. Pyripherals constructs the message using the address of the serial number register from the Registers.xslx spreadsheet. The message is then routed to the appropriate communication controller using the controller addresses defined in the ep_defines.v file and by the user code which provides the controller/bus name at initialization of the UID instance. Pyripherals formats messages for each type of communication protocol. In the user code example, the UID chip is connected to the I2CDAQ bus and has messages routed through the I2C0 communication controller such that the UID Python class inherits from a parent I2CController class.

**Table 1:** Each row shows a bit-field with a name and an address. This information is typically extracted from the datasheet of the peripheral chip. Addressable registers in a peripheral have multiple bits (often 16 or 32) that allow multiple data fields to be held in each register. To account for this, the location within a register is indicated by the high and low bit index columns. The default value of the register is stored to support verification of communication by checks of read-only registers.

Name	Hex Address	Default Value	Bit Width	Bit Index (High)	Bit Index (Low)
RESULT	0x00	0xf	12	11	0
CHAN	0x00	0x0	4	15	12
CONFIG	0x01	0xff	8	7	0
ID	0x01	0x0	8	15	8
